# Analytical considerations for accurately capturing the relevant species contributing to vitamin D status in liquid chromatography‐tandem mass spectrometry assays

**DOI:** 10.1002/ansa.202100057

**Published:** 2021-12-06

**Authors:** Dietrich A. Volmer, Caroline S. Stokes

**Affiliations:** ^1^ Bioanalytical Chemistry Institute of Chemistry Faculty of Mathematics and Natural Sciences Humboldt University Berlin Berlin Germany; ^2^ Food and Health Research Group Thaer‐Institute Faculty of Life Sciences Humboldt University Berlin Berlin Germany; ^3^ Department of Molecular Toxicology German Institute of Human Nutrition Potsdam‐Rehbrücke Germany

**Keywords:** 25‐hydroxyvitamin D, interferences, LC‐MS/MS, mass spectrometry, status marker, vitamin D

## Abstract

This tutorial review focuses on analytical challenges encountered with the liquid chromatography‐tandem mass spectrometry determination of 25‐hydroxyvitamin D, which is currently still considered the metabolite that is most representative of vitamin D status. It describes how multiple binding states of circulating 25‐hydroxyvitamin D (phase II metabolites, epimers, free/bioavailable/protein‐bound species) can influence the accuracy of the analytical determination. It also summarizes important chemical species that can inadvertently contribute to vitamin D status and thus cause systematic errors. These interfering endogenous and exogenous compounds might be isomers of vitamin D, constitutional isomers or isobars and the article outlines techniques to eliminate or minimize these interferences, including chromatographic separations, ion mobility spectrometry, and high‐resolution mass spectrometry.

## 1 INTRODUCTION

This tutorial review was prompted by a recent article by Jenkinson et al,^1^ who presented a novel method that permits simultaneous analysis of phase II metabolites of vitamin D_3_ (sulfates and glucuronides) and unconjugated circulating 25‐hydroxyvitamin D_3_ (25(OH)D_3_) using identical instrumental method conditions. The authors’ aim was to answer the question whether combined serum measurement of unconjugated and conjugated forms is better suited for assessing vitamin D status than current methods. Importantly, they demonstrated that sulfated conjugates form a significant proportion of the circulating vitamin D metabolites, whereas glucuronide conjugates are less important. The authors suggest that a combination of both conjugated and unconjugated measurements may provide a more accurate assessment of vitamin D status.^1^

Generally, there is an ongoing discussion over what exactly needs to be measured to properly assess vitamin D status.^2^, ^3^ Premer and Schulman recently posed the question “Have we been measuring the wrong form of vitamin D?”^2^ The question referred to the role of vitamin D in coronary artery disease, which has been under scrutiny, with unreliable results concerning its prognostic value and role in therapy. Moreover, recent studies on differences of vitamin D binding protein (DBP) and vitamin D levels in various populations have triggered the question of whether “technical errors” in using total rather than free/bioavailable 25(OH)D concentrations are the reasons for observed discrepancies.^3^

This article focuses only on the 25(OH)D metabolite, which is currently still considered the metabolite that is most representative of vitamin D status, and its assessment by liquid chromatography‐tandem mass spectrometry (LC‐MS/MS) assays (although many of the issues described here will equally apply to other analytical methods).^4^ Vitamin D status includes the total of two circulating vitamin D metabolites: 25(OH)D_2_ and 25(OH)D_3_. Herein, we will mostly refer to the 25(OH)D_3_ metabolite. This tutorial review will not discuss the physiological relevance of the various vitamin D species, their biological activities, or their suitability as improved vitamin D status markers. The present authors refer interested readers to an excellent overview of the “changing landscape” of vitamin D status assessment by Hermann et al, which summarizes the various relevant species, their measurement, and their potential use as an improved status marker.^5^

Instead, it will summarize important chemical species that might inadvertently contribute to vitamin D status in its current definition and thus cause systematic errors. These interfering species might be isomers of vitamin D (without being biologically active), constitutional isomers, or isobars that exhibit similar or identical physicochemical properties during analysis. In addition, a fraction of the circulating target 25(OH)D marker compound in the serum/plasma sample may be ‘hidden’ or masked by other endogenous components and thus unavailable in the analysis under the conditions used.

In the following text, we will focus on phase II metabolites, epimers, free/bioavailable/protein‐bound species, isomeric vitamin D metabolites as well as other endogenous and exogenous interferences.

## 2 PHASE II METABOLITES

**FIGURE 1:**
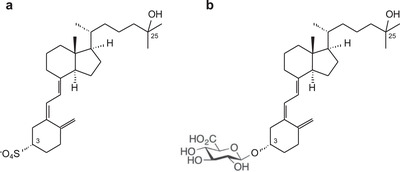
Structures of phase II metabolites of vitamin D: (a) 3‐sulfate; (b) 3‐glucuronide

We start the discussion with vitamin D conjugates (phase II metabolites: glucuronides and sulfates, Figure 1), which were, for the first time, measured together with the unconjugated (regular) 25(OH)D by Jenkinson et al.^1^ using a single assay under identical analytical LC‐MS/MS conditions. The authors added an enzymatic hydrolysis step during sample preparation, which released conjugated 25(OH)D into solution, providing an extract containing the sum of all 25(OH)D forms.

Jenkinson et al.^1^ highlight the potential importance of including the conjugated forms for status assessment (in particular the sulfate fraction) and suggest that it may constitute a circulating vitamin D binding protein‐associated reservoir for bioactive vitamin D.

Higashi et al. presented an LC‐MS/MS method that enables simultaneous analysis of conjugated vitamin D sulfate metabolites and unconjugated vitamin D metabolites in serum after derivatizing the vitamin D species with a Cookson‐type reagent, where the unconjugated forms remained intact.^6^ Alternatively, simultaneous analysis of 25(OH)D and its sulfated metabolite was achieved by using positive ionization for the unconjugated form and negative ionization for the sulfate metabolite.^7^ Finally, the combined analysis of phase II sulfates and conjugates of 25(OH)D (without the unconjugated form) in plasma and serum has been shown in studies by Gao et al^8^ and Huyn et al.^9^

In many applications, the sulfate conjugates are analyzed in a separate assay from 25(OH)D, however, utilizing, for example, the deprotonated molecules formed from the acidic sulfate group for sensitive detection in ESI negative ionization mode.^10^

## 3 EPIMERS

**FIGURE 2:**
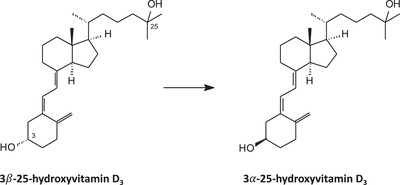
Epimerization pathway of 25(OH)D_3_

The parallel epimerization pathway of 25(OH)D provides epimerized vitamin D metabolites by reversal of the stereochemistry of −OH at C‐3 (β→α), which then also circulate in serum (we use the following nomenclature for the two 25(OH)D_3_ epimers: regular 25(OH)D_3_ = 3β‐25(OH)D_3_; 3‐epi‐25(OH)D = 3α‐25(OH)D_3_, Figure 2). Premature infants exhibit the highest 3α‐25(OH)D_3_ levels, with hepatic immaturity being suggested as a potential reason.^11^, ^12^ The metabolic products of 3α‐25(OH)D_3_ possess no or little calcemic activity, making analytical separation of 3α‐ and 3β‐25(OH)D_3_ obligatory, to avoid overestimating vitamin D status if both species are assessed together.^13^ In fact, the presence of the 3α epimer has been suggested to overestimate serum 25(OH)D_3_ concentrations by up to 25%.^14^ In adults, the 3β‐25(OH)D_3_ levels are usually much lower (<10%)^15^, ^16^ and it has sometimes been suggested that this contribution is negligible for adults, not requiring an LC‐MS/MS method that differentiates the two epimers, only for samples of patients younger than 1 year.^17^ For example, we previously reported a linear correlation between 3α‐25(OH)D_3_ levels and 25(OH)D_3_ in a cohort of 91 patients with chronic liver diseases.^13^ Overall, the findings supported previously published studies and the conclusion that the low concentrations of 3α‐25(OH)D_3_ are not likely to contribute significantly to vitamin D status for the majority of adults. We did, however, observe greater variability in patients with lower vitamin D status, particularly in those with very low 25(OH)D concentrations. Specifically, a significant (*P* < 0.001) difference in the relative contribution of 3α‐25(OH)D to total 25(OH)D in serum was noted based on the accepted 25(OH)D cut‐offs and categories of vitamin D status,^18^ with 2.4% contribution reported for normal vitamin D levels (≥30 ng/ml), 4.8% for patients in the insufficiency category (20‐29.9 ng/mL), 5.2% for those in the deficiency category (10‐19.9 ng/mL) and finally, patients with a severe vitamin D deficiency (< 10 ng/mL) displayed the highest relative contribution, with 5.8% reported for this group.^13^ From a clinical perspective, such interferences in patients with low circulating vitamin D levels could be crucial, because vitamin D status is used to guide the need for vitamin D replacement strategies. Thus, in such patients, the accurate quantification of vitamin D concentrations is paramount.

**FIGURE 3:**
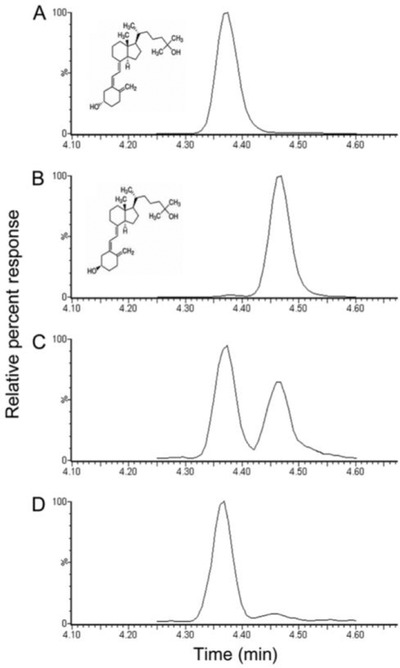
Chromatographic separation of 3α‐25(OH)D_3_ from regular 3β‐25(OH)D_3_. LC‐MS/MS chromatograms of injections of (a), pure 3β‐25(OH)D_3_ standard; (b), 3α‐25(OH)D_3_ standard; (c), infant serum sample containing 68.5 nmol/L 3β‐25(OH)D_3_ and 44.6% relative 3α‐25(OH)D_3_ concentration; (d), adult serum sample containing 79.0 nmol/L 3β‐25(OH)D_3_ and 4.2% relative 3α‐25(OH)D_3_ concentration. Reprinted with permission by Oxford University Press from ref. 15

From an analytical, LC–MS/MS point of view, this separation of the epimers cannot be achieved in the mass analyzers because (a) these species are isomers with the same exact mass and (b) the collision‐induced dissociation (CID) mass spectra of both epimers are virtually identical.^19^ The 3α epimer can be readily separated from 3β‐25(OH)D by chromatography, however, and many assays have been described showing baseline resolution of the two epimers^15^, ^20^ (Figure 3).

Some promising work on ion mobility spectrometry (IMS) has recently been shown by Yost and coworkers, who were able to separate the two 25(OH)D epimers in the IMS device prior to mass spectrometry‐based on the mono‐sodiated species.^21^

Incidentally, 3α concentrations can be quantitively captured separately if required, but proper isotope standards must be used for both epimer species to avoid calibration errors, as both species exhibit different response behaviors.^19^, ^22^, ^23^ Alternatively, a response levelling derivatization strategy could be implemented.^13^

## 4 FREE VITAMIN D, BIOAVAILABLE VITAMIN D, PROTEIN‐BOUND VITAMIN D

For classical status marker assays using LC‐MS/MS, the existence of different binding states of vitamin D, unbound or bound to proteins, is of little importance for accurate assessment of vitamin D status, as long as 25(OH)D is fully released from the protein during sample preparation. Since this is achieved in virtually all LC‐MS/MS assays, free and protein‐bound vitamin D are measured together from the same sample preparation extract.

However, if free vitamin D or bioavailable vitamin D are to be measured individually, the sample preparation step must allow the separation of free 25(OH)D from the bound fraction.

The unbound 25(OH)D is hypothesized to play an important role in the numerous non‐classical actions of vitamin D. As reflected in the “free hormone hypothesis,” it is the free 25(OH)D metabolite that is able to enter cells, thus exerting biological actions.^24^, ^25^ The clinical relevance of these metabolites remains to be unraveled; however, in order to do this, accurate quantification methods need to become widely available.

For further information in this area of research, the present authors refer the interested reader to, for example, refs. ^24^, ^25^, ^26^, ^27^, ^28^.

## 5 OTHER ISOMERIC VITAMIN D METABOLITES

Tuckey *et al*. recently^29^ reviewed the different enzymes that act on the various vitamin D molecules and described a large number of relevant vitamin D‐transformation products that have been detected or can be expected in biological samples. For example, the authors describe two mono‐hydroxylated species, 20*S*(OH)D_3_ and 22(OH)D_3_, as a result of CYP11A1 action on vitamin D_3_, with concentrations reported at 2.9 nM and 6.0 nM, which are comparable to the 3α‐25(OH)D_3_ epimer levels.^29^ Similarly, 1α‐hydroxyvitamin D, which is a synthetic prodrug, has been suggested as isomeric interferences.^14^

If the chosen chromatography conditions do not separate these isomeric compounds from 25(OH)D, they will be co‐measured with the status marker, hidden under the 25(OH)D peak. As isomers, they have the same exact mass and thus HRMS in full scan mode cannot distinguish them; even MS/MS provides virtually similar CID spectra for most vitamin D molecules.

Isomeric forms of 25(OH)D are also formed by thermal isomerization of 25(OH)D and these reactions then reduce the amount of 25(OH)D in solution. As recently shown by Bedner and Lippa,^30^ to avoid conversion of 25(OH)D species to their pre‐25(OH)D forms, appropriate handling and storage conditions are required, with a suggestion of storing samples at ‐20°C or colder. Nevertheless, even when samples are stored at low temperatures, special care must be taken to ensure reliable calibrations, as low temperatures cannot be maintained during all sample handling steps. The authors demonstrate^30^ that 1% pre‐25(OH)D_3_ equilibrium level is reached within only 5 h in a matrix‐matched calibration solution.

Finally, it remains to be seen whether ion mobility spectrometry separations may provide sufficient selectivity and resolving power in the future to separate hydroxylated vitamin D isomers prior to mass spectrometry.

## 6 ENDOGENOUS AND EXOGENOUS INTERFERENCES

**FIGURE 4:**
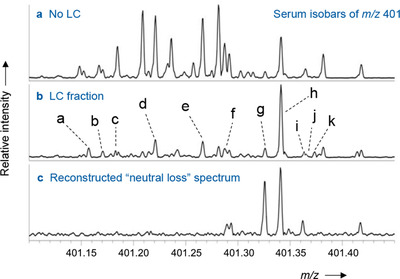
High‐resolution FTICR mass spectra of the (M+H)^+^ region of 25(OH)D_3_ at *m/z* 401 (area from *m/z* 401.10‐401.45), measured from a human serum extract: (a) serum extract showing all isobars in the sample extract; (b) fraction of the chromatogram showing all isobars that co‐elute with 25(OH)D (peak h); (c) ‘neutral loss scan’ calculated from the CID data using the *m/z* 383 product ions to reconstruct an artificial mass spectrum of all precursor ions that exhibit H_2_O loss upon CID. Peak assignments: a‐f, isobars of unknown structure; g pentaerythritol oleate (PEO), h 25 hydroxyvitamin D_3_; i‐k, isobars of unknown structures. The elemental formulae of all unknown isobars are given in ref. 31. Reprinted with permission by John Wiley & Sons from ref. 31

**FIGURE 5:**
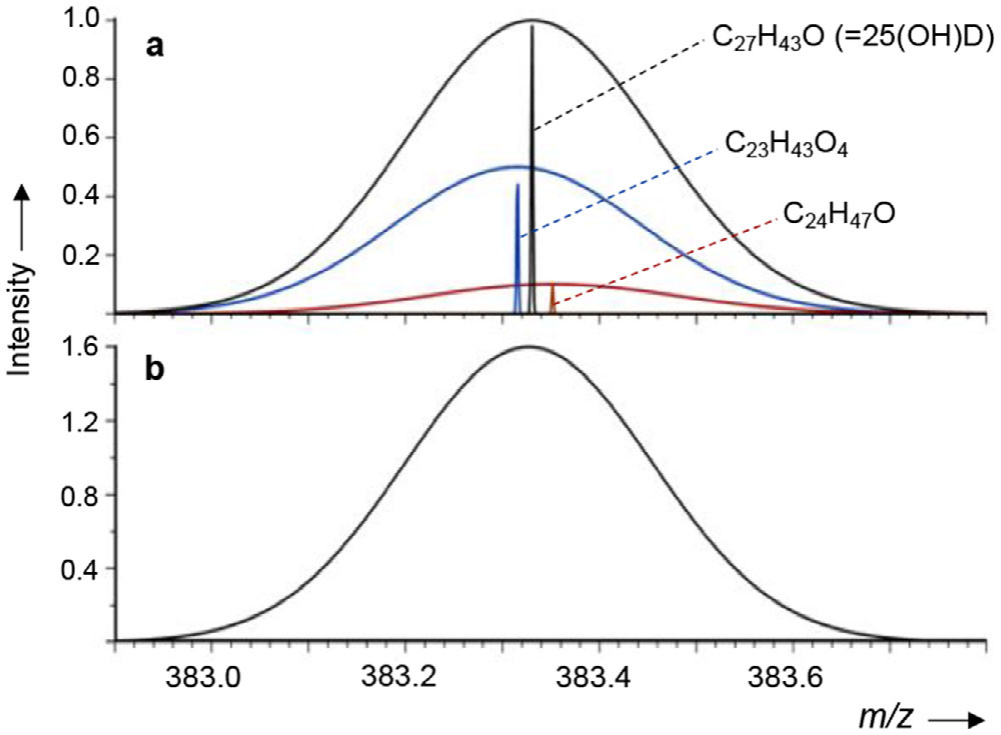
Simulated overlap of isobaric [M+H–H2O] product ion peaks at *m/z* 383 in the CID spectrum of 25(OH)D_3_ (after activation of [M+H] at *m/z* 401) using (a) high‐resolution FTICR and (b) low resolution QqQ instruments. The full‐ width‐at‐half‐maximum (FWHM) values for QqQ and FTICR were 0.3 and 0.002 u, respectively. The area‐under‐the‐curve for the QqQ signal was 1.6× larger than that of the isolated isobar for 25(OH)D on the FTICR instrument. Reprinted with permission by John Wiley & Sons from ref. 31

**FIGURE 6:**
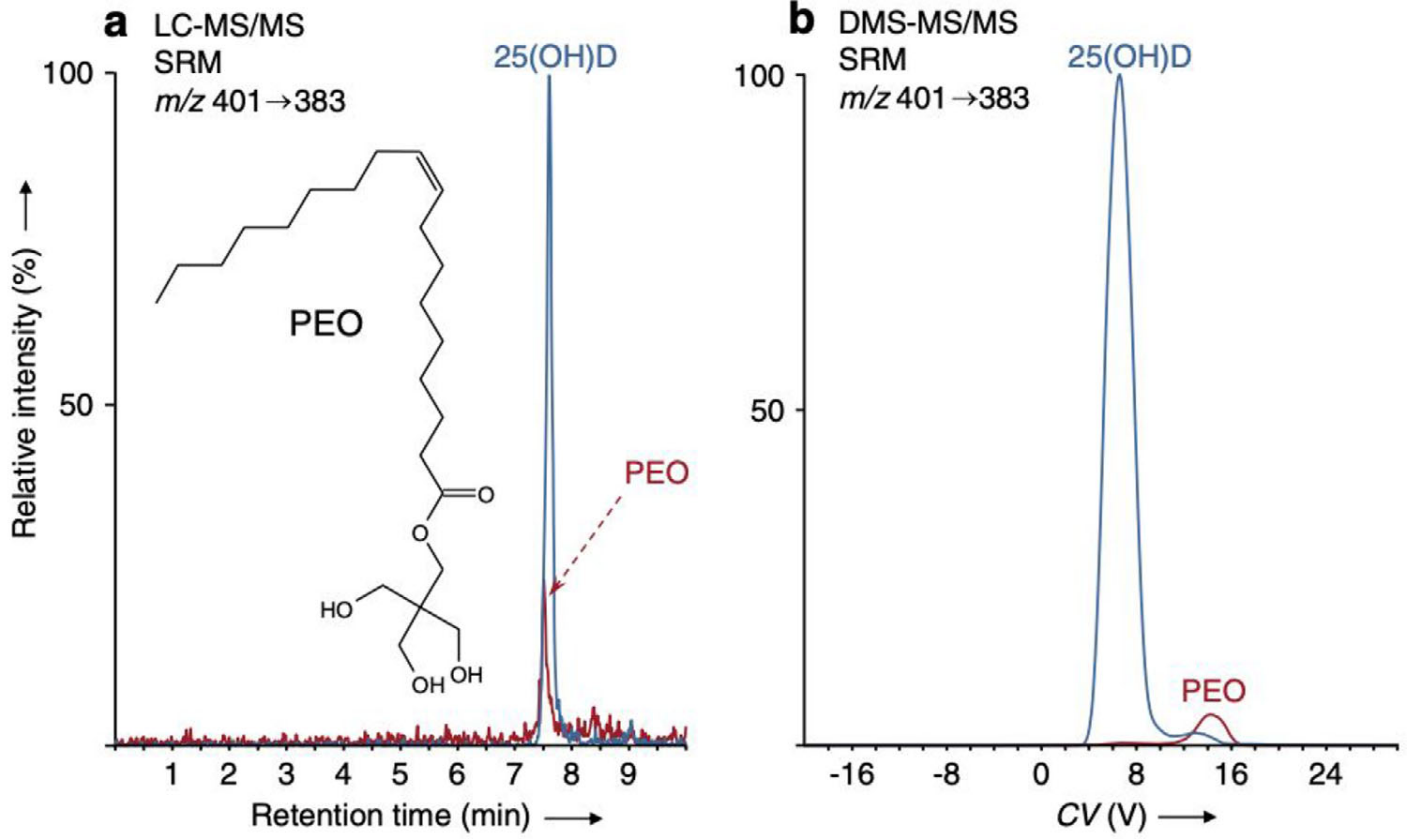
(a) LC‐MS/MS and (b) LC‐DMS‐MS/MS separations of 25(OH)D and pentaerythritol oleate (PEO). MRM traces correspond to *m/z* 401 → 383 transitions after individual injections of equimolar amounts of pure standards of the two compounds. Reprinted with permission by John Wiley & Sons from ref. 31

Many issues of method selectivity in LC–MS/MS of vitamin D from serum/plasma originate from non‐vitamin D compounds in the sample, which generate isobaric and isomeric ions with the potential to interfere in both MS and MS/MS modes. Many LC–MS/MS assays for 25(OH)D performed on triple quadrupole MS use unspecific MRM transitions, which provide interference even in the MS/MS domain.^4^ The theoretical “isobaric space” of potential interferences in biological samples is very large, as demonstrated e.g. from the METLIN metabolite database.^4^ Moreover, detailed ultra‐high resolution FTICR MS analysis of the immediate area around the precursor ion mass of 25(OH)D_3_ from a serum sample demonstrated the presence of multiple isobaric ions, several of which also generated significant ion currents in the MRM transition (Figure 4).^31^ These compounds have the potential to increase the peak area of 25(OH)D_3_ and cause a systematic error (Figure 5). In the present authors’ analysis, one particular exogenous compound, pentaerythritol oleate (PEO), which is a technical lubricant, fully co‐eluted with 25(OH)D and caused the peak area to increase by approximately 20% across a range of patient serum samples.^31^ This molecule may have leached from a seal of the chromatography system. It was possible to eliminate this interference (and many other isobars) by using LC‐DMS‐MS/MS (DMS = differential ion mobility spectrometry) (Figure 6). DMS, similar to IMS mentioned in the previous section, separates molecules according to their conformation rather than mass.

Maunsell et al considered the endogenous bile acid precursor 7α‐hydroxy‐4‐cholestene‐3‐one as interference that potentially contributes to vitamin D status if not separated by chromatography.^32^ Similarly, the toxic oxysterol 7‐ketocholesterol would, in theory, interfere with 25(OH)D if not resolved.^31^ Both compounds are constitutional isomers of 25(OH)D and will exhibit very similar behavior to 25(OH)D under MS/MS conditions and, of course, have identical exact masses and are thus impossible to resolve via their exact mass by HRMS. Carter emphasizes^33^; however, that there is no indication for a strong contribution of 7α‐hydroxy‐4‐cholestene‐3‐one in routine LC‐MS/MS assays. Furthermore, in our work, we have shown that 7α‐hydroxy‐4‐cholestene‐3‐one and 7‐ketocholesterol were readily separated from 25(OH)D by liquid chromatography using a conventional C‐18 column. Furthermore, the potential endogenous isobar 1,2‐didecanoyl‐sn‐glycerol was equally easy to separate from 25(OH)D by LC.^31^

Importantly, there are many other endogenous constitutional isomers of 25(OH)D such as the oxysterols 24‐hydroxy‐7‐dehydrocholesterol and 25‐hydroxy‐7‐dehydrocholesterol,^34^ which have the potential to interfere with 25(OH)D in triple quadrupole assays using an unspecific MRM transition. Similarly, HRMS would require a specific fragment ion after MS/MS experiments to distinguish them from 25(OH)D, which may be difficult because of the typical “picket fence” series of decompositions seen in CID spectra of these compounds at higher collision energies.^4^

For quantitative HRMS applications, unresolved isobaric interferences must often also be eliminated using MS/MS. We have recently shown^35^ that even the tail‐end of an almost fully resolved peak in a full‐scan QqTOF analysis of 25(OH)D in serum introduced isobaric crosstalk and thus systematic errors during the analysis, which made the use of MS/MS data acquisition necessary.

Of course, liquid chromatography will remove many of the interfering isobars and isomers. Nevertheless, because of the large number of possible endogenous and exogenous metabolites, many of which have the potential to mimic the mass spectral behavior of 25(OH)D,^4^ it is very likely that multiple isobaric interferences will still co‐elute with 25(OH)D.

## 7 CONCLUSIONS

This tutorial has addressed two areas of concern with analytical assays for quantification of the 25(OH)D status marker, with particular emphasis on LC‐MS/MS assays. First, it described how multiple binding states of circulating 25(OH)D can be captured in a single assay, namely protein‐bound 25(OH)D and conjugated forms of the status marker, in particular glucorinated and sulfated states. These can be determined in separate assays or combined, as outlined in the tutorial. Second, interferences have been explained that stem from other endogenous and exogenous compounds present in the sample extract. The elimination of these of isobaric and isomeric interferences currently heavily relies on proper chromatographic separation, supported by high resolving power mass analyzers if available.

Interferences from ion suppression effects have not been discussed in this tutorial review, as they are usually corrected by using stable isotope standards of 25(OH)D. Ion suppression effects do not create systematic errors as long as the degree of protein binding of 25(OH)D and the isotope standard is identical.^36^

However, an often‐overlooked general issue in quantitative bioanalytical LC–MS/MS assays is the interference that the stable isotope standard might experience. The stable isotope standard's mass is usually 3‐6 Da higher than the precursor ion of the analyte and the level of interference may be different from the analyte, which would then can lead to systematic errors. HRMS assays with sufficient resolution are expected to reduce these effects but cannot address isomeric interferences.

In conclusion, since one never knows in advance, which exact endogenous and exogenous interferences (isobars and isomers) are present in a sample, only liquid chromatography and high‐resolution mass spectrometry, both with sufficient resolving power, can ensure analyses free of systematic errors. Of course, very often standardized methods are used to allow comparison of the measured vitamin D concentrations across different laboratories, in larger studies, and over long time periods, thus limiting the scope of tweaking the assays for maximum performance. The remaining systematic errors are then accepted (“two wrongs make it right”) as otherwise comparison of data is impossible.

## AUTHOR CONTRIBUTION

The manuscript was written through contributions of all authors.

## CONFLICT OF INTEREST

The authors declare no competing financial and nonfinancial interests. DAV is Editor‐in‐Chief of Analytical Science Advances.

## Data Availability

The data that are used in this review paper are available in the text and figures of this article.
